# The Distribution and Density of Water Mice (*Xeromys myoides*) in the Maroochy River of Southeast Queensland, Australia

**DOI:** 10.1371/journal.pone.0146133

**Published:** 2016-01-20

**Authors:** Janina Kaluza, R. Lesley Donald, Ian C. Gynther, Luke K-P. Leung, Benjamin L. Allen

**Affiliations:** 1 The University of Queensland, School of Agriculture and Food Sciences, Gatton, Queensland, 4343, Australia; 2 Queensland Parks and Wildlife Service, Department of National Parks, Sport and Racing, Maroochydore, Queensland, 4558, Australia; 3 Threatened Species Unit, Department of Environment and Heritage Protection, Bellbowrie, Queensland, 4070, Australia; 4 The University of Southern Queensland, Institute for Agriculture and the Environment, Toowoomba, Queensland, 4350, Australia; Australian National University, AUSTRALIA

## Abstract

The water mouse is a small and vulnerable rodent present in coastal areas of south-west Papua New Guinea, and eastern Queensland and the Northern Territory of Australia. Current knowledge regarding the distribution of the water mouse is incomplete and the loss of one local population has been documented in southeast Queensland, a region where pressures from urban and industrial development are increasing. Water mouse populations have not been studied intensively enough to enable the primary factors responsible for the local decline to be identified. We surveyed the distribution and density of the water mouse along the Maroochy River of southeast Queensland, near the southern extent of the species’ range, to gather baseline data that may prove valuable for detecting any future decline in this population’s size or health. All areas of suitable habitat were surveyed on foot or by kayak or boat over a three-year period. We found 180 water mouse nests, of which ~94% were active. Permanent camera monitoring of one nest and limited supplementary live trapping suggested that up to three individual mice occupied active nests. Water mouse density was estimated to be 0.44 per hectare of suitable habitat along the Maroochy River. Should future monitoring reveal an adverse change in the water mouse population on the Maroochy River, a concerted effort should be made to identify contributing factors and address proximate reasons for the decline.

## Introduction

Coastal wetlands are critical points of connectivity between terrestrial and marine ecosystems, and their environmental health is important for the proper functioning of both. Worldwide, coastal wetlands are threatened by a variety of factors including land use change, increasing human presence, invasive species and climate change [1,2]. These processes can manifest themselves as changes in species distribution, abundance and/or behaviour [3]. Mitigating the effects of these threatening processes on wetland species requires the ongoing collection of information useful for enabling best-practice management of species of conservation concern.

The east coast of Queensland, north-eastern Australia, is ~7,000 km long and is bordered by the Great Barrier Reef marine ecosystem and the tropical and subtropical terrestrial ecosystems of the Great Dividing Range. Along the coast, approximately 36 major rivers flow east into the Pacific Ocean from this Range. These contribute to a substantial number of coastal wetlands, which provide breeding grounds for many marine microorganisms, crustaceans, birds, fish and other species [4]. Queensland is home to over 4.7 million people, 85% of whom live within 50 km of the coast along many of these rivers (www.abs.gov.au). This human presence may have previously altered any balance between natural ecosystem processes and extant fauna at wetland sites. As we continue to explore the types of impacts humans may have on the environment, consistent research is required to determine any declining range of coastal fauna since European occupation of Australia in the late 1700s.

**Fig 1:**
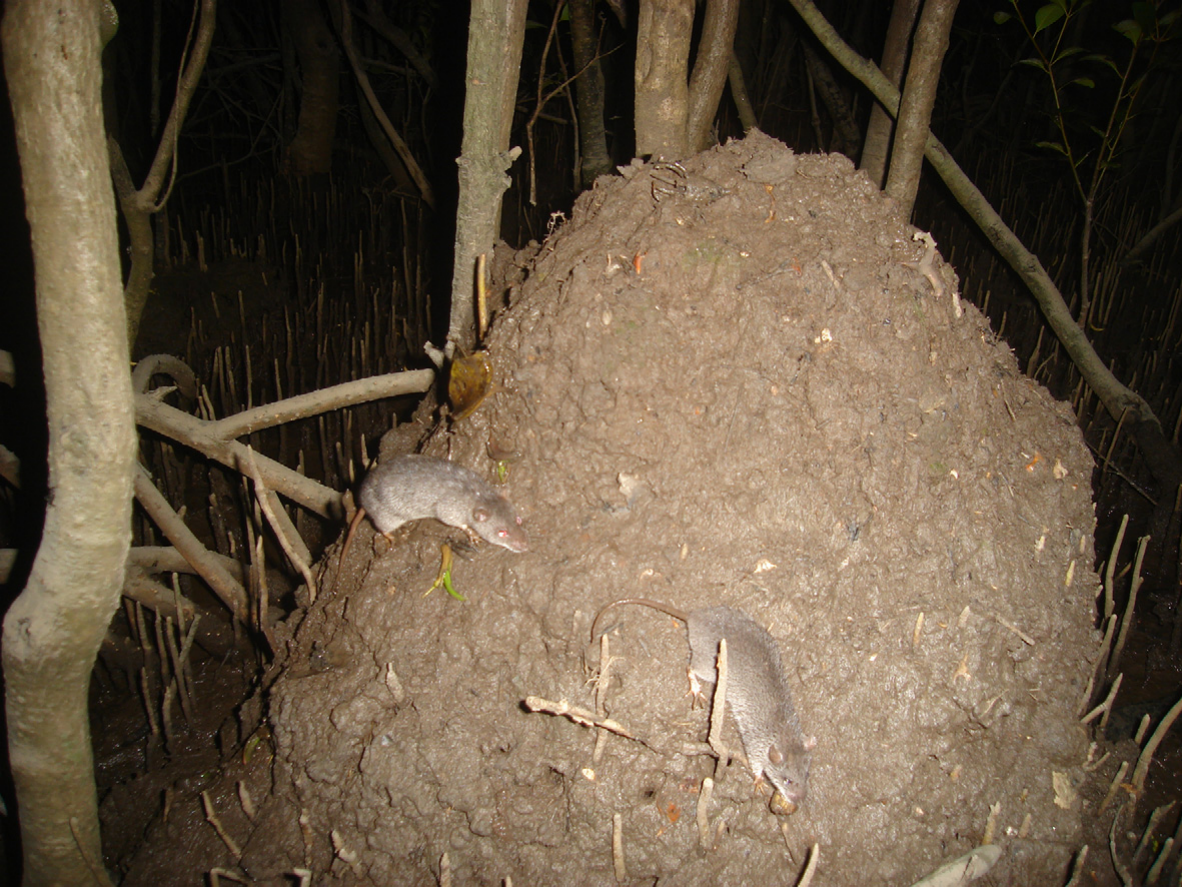
Two water mice maintaining a mound-style nest in a mangrove vegetation community of Sector 3 of the Maroochy River system, 8^th^ March 2012 (Photo: Janina Kaluza).

The water mouse (*Xeromys myoides*; also known as the false water rat or ‘yirrkoo’) is a small carnivorous rodent (~40 g) that builds and occupies elaborate nest structures in intertidal zones dominated by mangrove (e.g. *Avicennia marina* var. *australasica*, *Bruguiera gymnorhiza*, *Aegiceras corniculatum*, *Rhizophora stylosa*, *Excoecaria agallocha*) and saltmarsh (e.g. *Enchylaena tomentose* var. *glabra*, *Sarcocornia quinqueflora*, *Sporobolus virginicus*, *Isolepis nodosa*, *Juncus kraussii)* vegetation communities (Fig 1; [5,6]), which are preferred habitat for this species. The distribution of the water mouse is currently known to extend from Papua New Guinea to the north coast of Australia and in eastern coastal wetlands as far south as the Gold Coast in southeast Queensland [7,8]. The Maroochy River is approximately 135 km from the southern edge of the species’ known range. Water mouse populations are believed to have become locally extinct from the Coomera River over the last few decades [7,9]. The ecological roles of the water mouse are not clear. However, as one of the few native terrestrial mammals occupying these wetlands, it is likely to be an important predator of molluscs and crustaceans, prey for nocturnal raptors, reptiles and other species [10], and potentially provides ecosystem services for other native animals through the construction of mud nests that represent small islands in the intertidal zone.

In this study, we describe the distribution and density of water mice in the Maroochy River system of southeast Queensland. Our aim was to generate baseline population data that may be useful for determining trends in distribution and abundance following future monitoring of the species.

## Methods

### Ethics statement

Water mice are protected and presently listed as ‘vulnerable’ under the Federal *Environment Protection and Biodiversity Conservation Act (1999)*, and are supported by a national recovery plan [11]. Permission to enter the study site was granted by the Queensland Parks and Wildlife Service and Marine Parks Authority. The Animal Ethics Committee of the Department of Agriculture, Forestry and Fisheries (DAFF) approved this study (permit approval number: CA 2014/08/797), and the project was carried out in accordance with this approval.

### Study site

**Fig 2:**
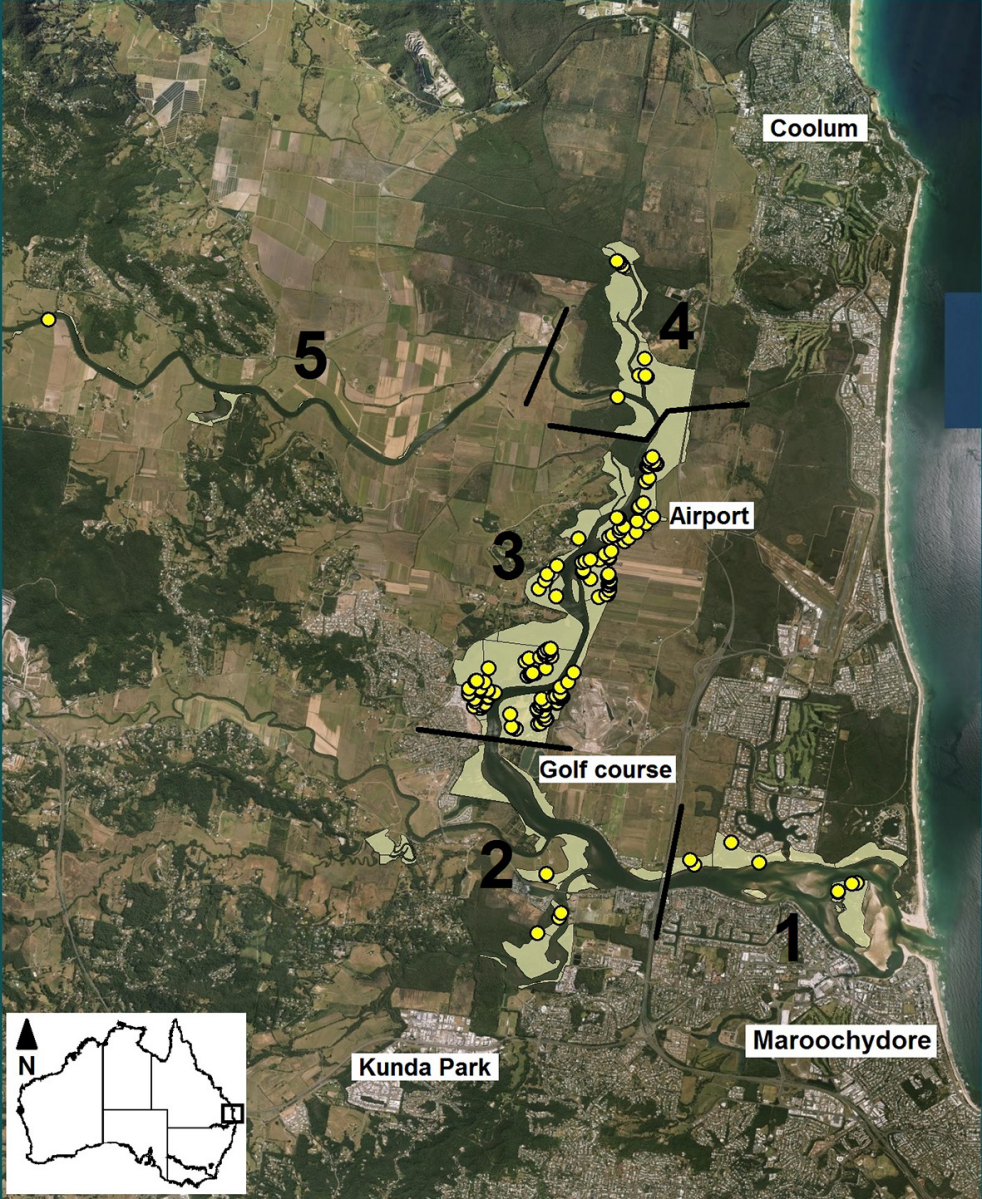
The study site on the lower Maroochy River of Queensland’s Sunshine Coast, showing the location of 180 water mouse nests (yellow circles) and areas of suitable water mouse habitat (shaded areas). Numbering indicates survey sectors. Map was created new by the authors in ArcGIS v10.1 (ESRI Inc.).

The Maroochy River is located on the Sunshine Coast of southeast Queensland. It is a popular and growing residential area with >250,000 people [12]. The area is subtropical, with a warm and humid climate, and receives an average of ~1,550 mm of rainfall annually, which peaks in summer (www.bom.gov.au). The area surrounding the Maroochy River supports both natural and human-modified areas (Fig 2). The former includes paperbark (*Melaleuca* spp.) swampland, open forest communities and small fragments of subtropical rainforest, with mangrove and saltmarsh fragments adjoining the river’s edge in many places. Human land use around the river is predominantly agricultural (sugar cane crops and ex-sugar cane areas now supporting grassland) and urban residential (Fig 2). The area is undergoing substantial development, including new golf courses, industrial areas, residential areas and an extension to a local major airport.

### Water mouse distribution and abundance

For the purposes of our surveys, the lower Maroochy River was arbitrarily divided into five adjoining sectors based on unique features of the river system, such as bends and tributaries. We then systematically identified all suitable water mouse habitat in each sector using high-resolution aerial photography and GIS vegetation datasets maintained by the Queensland Herbarium. We then undertook extensive ground surveys for water mouse nests within these sectors between September 2011 and December 2014. All suitable habitats were surveyed by boat, kayak, or typically on foot during low tide, on multiple occasions for some areas, to minimise the possibility of overlooking nests. Nests were classified as ‘active’ or ‘inactive’ based on sign of recent water mouse activity (e.g. fresh foot prints, mud daubing, or the presence of fresh prey remains). Absolute density of nests was calculated as the number of nests per hectare of suitable habitat.

Three automated trail cameras (Pixcontroller trail cameras, Digital Eye TM, CAMO60 6.0, Digital Trail Camera) were deployed between March 2012 and December 2014 at one mound-style nest (Fig 1) in Sector 3 in a longitudinal study designed to determine the number of individual water mice occupying the nest. Mound-style nests are the most common form of water mouse nests at this site, and this nest was of broadly similar construction to the others (N. Kaluza, unpublished data). Live trapping (under Queensland Department of Agriculture and Fisheries Animal Ethics Committee approval number SA 2013/12/452 to IG) was undertaken on one occasion on the night of the 4^th^ June 2014, using a total of 75 Elliott traps (for more information on Elliot trapping, see [13]) set around this nest and two nearby nests on a supralittoral bank (i.e. a low bank at the boundary between the intertidal and terrestrial communities) less than 100 m away. We used a barricade trapping approach, whereby the nests were first completely surrounded by a flywire mesh fence installed at least 50 mm below ground level and 250 mm above ground. Multiple Elliott traps were then placed both inside (N = 16–18) and outside (N = 8–9) the barricade with the aim of catching water mice that were inside the nest and those that were absent from the nest at the time the barricade was established. Each trap was baited with a piece of blue pilchard (*Sardinops sagax*) approximately 3 cm in length.

Traps were checked multiple times throughout the night to ensure they were not inundated as the tide rose. Captured animals were removed from traps, sexed, measured and then released at the point of capture at midnight and/or dawn. Captured animals were temporarily marked by clipping a small patch of hair on the crown with scissors; individual identification was based on the unique patterns of white spots on the dorsal pelage. A small ear snip and a saliva sample were collected, and body weight, head length, head and body length, tail length, ear length and hindfoot length were measured. Ear and saliva samples were given to the Queensland Museum for specimen cataloguing and keeping. Age and reproductive condition were also assessed. At no time were animals anaesthetised. Trapping results were used to attempt to verify the number of individuals within an active nest, as recorded by camera.

## Results

**Fig 3:**
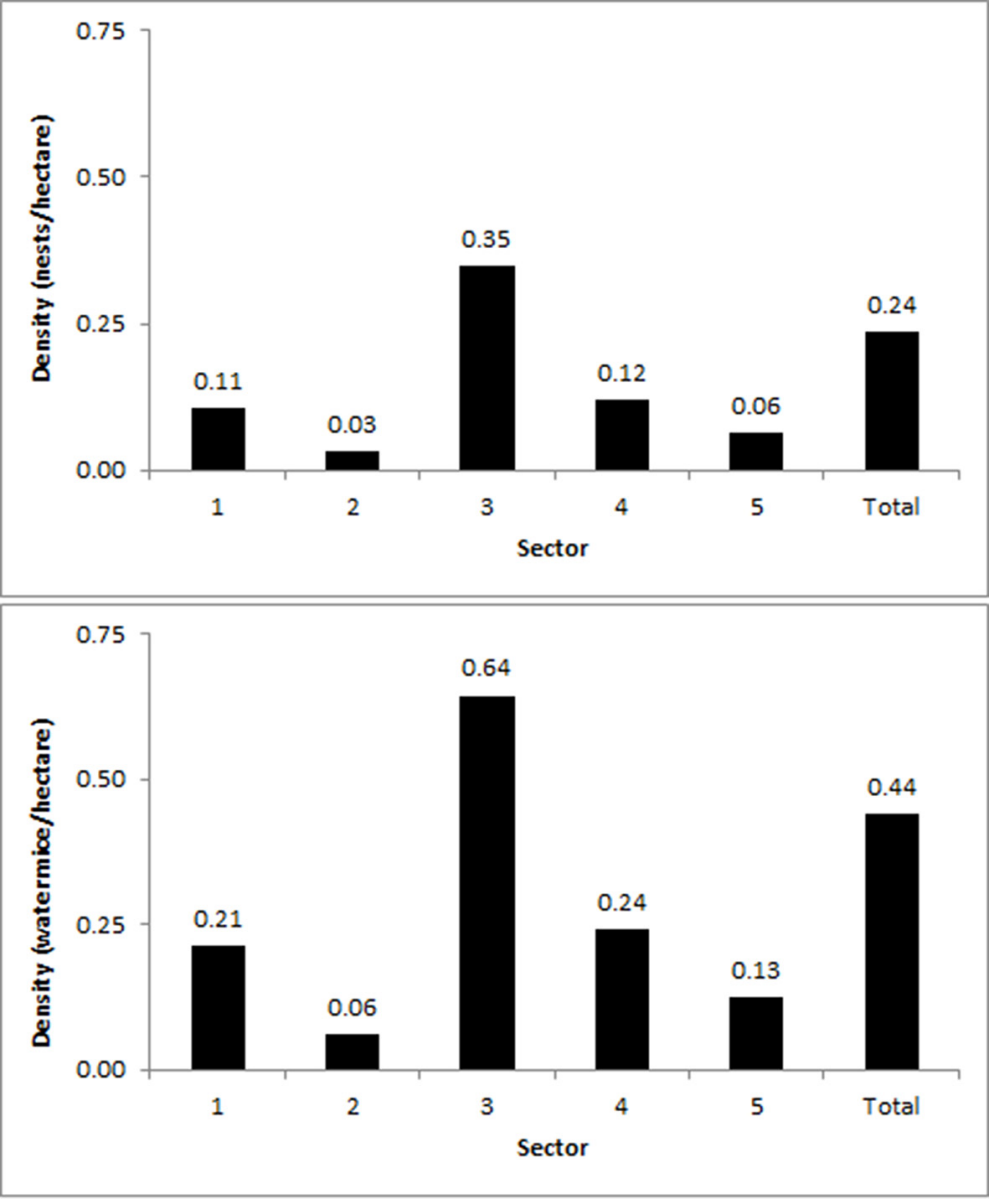
The density of active and inactive water mouse nests (top) and water mouse individuals (assuming an occupancy rate of two mice per nest; bottom) in suitable habitat across all survey sectors along the lower Maroochy River.

We identified a total of 765 ha of suitable water mouse habitat in the lower Maroochy River, most of which was in Sector 3 (Fig 2). Approximately 600 ha of land along the river system is designated and managed by state and local governments as conservation reserves; ~23% of water mouse habitat (~175 ha) occurred within these reserves. We located a total of 169 active and 11 inactive water mouse nests. Most of the active and all the inactive nests were in Sector 3 (Fig 2, Table 1), and 53 nests occurred within reserves. The absolute density of active nests across all five survey sectors was 0.22 nests per hectare of suitable habitat (Fig 3).

**Table 1:** The number of water mice nests in the lower Maroochy River.

Sector	No. of active nests	No. of inactive nests	Total no. of nests
1	11	0	11
2	3	0	3
3	140	11	151
4	14	0	14
5	1	0	1
**Total**	**169**	**11**	**180**

The trail cameras recorded c. 10,000 photos. Of these, c. 8,000 photos captured water mice. No more than two individual water mice were observed in any one photo. However, body size, pelage, and the sequence and timing of behaviours observed in some photos suggested that up to three individual water mice (two adults and a juvenile) used the nest under surveillance at any one time during the course of the study.

Live trapping yielded five individual water mice from two nests–three individuals were captured inside the barricade fence at one of the supralittoral bank nests and two individuals (one inside the barricade fence and one outside) were caught at the nest that was monitored by the trail cameras (Fig 1); no water mice were captured at the third nest (Table 2). Despite these trapping results, we cannot be sure that any individual captured inside the barricade fence at a particular nest had occupied the nest in question because of the possibility that water mice may have traversed the fence barrier via subterranean tunnels. This reduces the reliability of any conclusions drawn about numbers of individual occupying nests based solely on the trapping data. Nevertheless, assuming that two individuals occupy an active nest, water mouse density in the study area was 0.44 individuals per hectare of suitable habitat (Fig 2), suggesting a local population size of ~340 individuals. Assuming that three individuals occupy each active nest, water mouse density would be as high as 0.66 individuals per hectare, or ~500 individuals in the lower Maroochy River.

**Table 2:** The number of individual water mice trapped around nest sites at midnight and dawn on 4–5 June 2014, in the Maroochy River system.

	Midnight	Dawn		Combined
Nest	New captures	New captures	Recaptures	Total no. of individuals
1	3^	0	3^	3
2	0	0	0	0
3	1^	1*	1^	2

^ captured inside the barricade

* captured outside the barricade

## Discussion

This study is the first to report the distribution and density of water mice in the Maroochy River. Given that the species has become locally extinct from one site in a similar river system in southeast Queensland [9], the 180 nests we located (Fig 2, Table 1) and the ~340–500 individuals likely to be present in this estuarine system represent a population of considerable conservation significance. By comparison, our nest tally is equivalent to the total number of nests found during an intensive, two-year survey of the full extent of the Great Sandy Strait [14]–a Ramsar and Marine Park site recognised as supporting a water mouse population of national importance [11]–even though we surveyed a much smaller area along the Maroochy River. No other detailed studies of this species has identified such high numbers or densities of nesting structures associated with any water mouse population (e.g. [6,10,15]).

Although a large range of threats to the water mouse across the species’ range has been identified (e.g. [5,7,8,11]), precise threats to the wetland communities along the Maroochy River are yet to be established. Nevertheless, they are likely to include the direct and indirect effects of rapid land use change from natural ecosystems to agricultural, residential and industrial areas. Extant mangrove habitats are highly fragmented ([16]; Fig 2), and for over 100 years the adjacent crop farms have used substantial quantities of pesticide, herbicide and fertilizer [17]. Minimal buffer zones exist between wetlands and agricultural areas, which are typically located side by side (Fig 2). Historically, major earthworks (to improve drainage for agriculture) have allowed runoff to be directed straight into the adjacent wetlands [18]. Draining of wetlands and land reclamation for agriculture and development have also occurred. This alteration of the environment not only removes wetlands completely, but is also likely to have affected remaining wetlands through changes to salinity and sediment levels [19] which, in turn, affect the abundance of crustaceans and other prey species for water mice [11,20].

Results of the current study imply that the water mouse has persisted along the Maroochy River despite these historical changes brought about by the conversion of natural areas to agricultural land. However, as current land use undergoes further rapid change from agricultural to residential with much higher human densities, the nutrient-enriched soils of the former cropping land become disturbed and exposed to runoff during construction, and increased stormwater flows and pollutants after construction [20,21]. Little is known about the chemical and physical composition of mud required to bind water mouse nests [11,14,22], the resources required to sustain water mouse populations, the processes that negatively affect those resources, or threshold levels of environmental change that water mice (or their prey) may be able to withstand [1,11].

Water mouse nests were not evenly distributed throughout the suitable habitat available to them, but were instead clumped (Figs 2 and 3), suggesting that factors other than habitat availability per se may influence the species’ local distribution and density. A very cautious approach to land use change in this area is warranted to protect the water mouse and other species from local extinction [12,17,21–23].

We can be confident that our figure for absolute nest density accurately reflects the true situation given the extensive surveys that were conducted. We cannot dismiss the possibility that some additional water mice nests may be present, although we believe there is unlikely to be a substantial number of nests not detected by our surveys. We are also confident in our population estimate of ~340–500 individual water mice in the lower Maroochy River. However, we acknowledge the limited data we have on the number of individuals occupying nests, and whether or not multiple nests are shared by individuals or groups–factors that contribute to accurately assessing the abundance and density of individuals. Van Dyck [10] previously recorded up to eight individuals per nest on Stradbroke Island; if this were the case in our study area, then water mouse density would be as high as 1.76 mice per hectare, or as many as ~1,350 mice in the lower Maroochy River. Alternatively, if the same individuals or groups use multiple nests, water mouse abundance and density will be lower than our estimates. A greater understanding of nest occupancy, demography, and reproductive and movement behaviour is logically the next step for assessing the true conservation status of the water mouse population in this area.

The recent and local extinction of a water mouse population within the region [9] is a part of the current trend of mammal declines in Australia [7,24]. Further such extinctions of water mouse populations in southeast Queensland and elsewhere across the species’ range must be avoided. If robust population monitoring practices can be successfully implemented and maintained, researchers may be able to identify the threats and causes of any local water mouse decline, potentially allowing the species to be recovered. In turn, this may lead to a greater understanding of how to better manage and conserve other coastal wetlands and their constituent wildlife in the face of increasing global threats and species declines.
